# Insight into Point Defects and Complex Defects in β-Mo_2_C and Carbide Evolution from First Principles

**DOI:** 10.3390/ma15134719

**Published:** 2022-07-05

**Authors:** Jing Guo, Yunli Feng, Cong Tang, Li Wang, Xiaoliang Qing, Qingxiang Yang, Xuejun Ren

**Affiliations:** 1 School of Engineering, Faculty of Engineering and Technology, Liverpool John Moores University, Liverpool L3 3AF, UK; 2015projectljmu@gmail.com (C.T.); x.qing@2020.ljmu.ac.uk (X.Q.); 2 Hebei Key Laboratory of Modern Metallurgy Technology, College of Metallurgy and Energy, North China University of Science and Technology, Tangshan 063009, China; yunlifeng1989@163.com; 3 School of Engineering and Materials, Queen Mary University of London, London E1 4NS, UK; l.i.wang@qmul.ac.uk; 4 State Key Laboratory of Metastable Materials Science and Technology, Yanshan University, Qinhuangdao 066004, China; qxyang@ysu.edu.cn

**Keywords:** point defect, β-molybdenum carbides (Mo_2_C), first-principles calculation, defect combination, formation energy, phase transition, phase engineering

## Abstract

In this paper, first principles method was adopted to investigate the point defects, Vanadium-related defects and defect combinations (vacancy (V), substitutional (S) and/or interstitial (I)) in molybdenum β-Mo_2_C and explore the use of first principles calculation data in analysing the link between different carbides and the effects of doping elements. Supercell models with different defect types were established and optimized, and the formation energy data of defects was developed. The structure evolution during the optimization process is analysed in detail to establish the main characteristics of changes and the relevant electronic properties. The data for different types of intrinsic defects and combined defects complexes was developed and key results is analysed. The results show that carbon vacancy (V_C_) is stable but does not inevitably exist in pure β-Mo_2_C. Interstitial site II is a very unstable position for any type of atoms (Mo, V and C), and analysis of the structure evolution shows that the atom always moves to the interface area near the interstitial site I between two layers. In particular, a C atom can expand the lattice structure when it exists between the layer interfaces. One type of the defects studied, the substitution of Mo with V (designated as ‘S_V-Mo_’), is the most stable defect among all single point defects. The data for defect complexes shows that the combination of multiple S_V-Mo_ defects in the super cell being more stable than the combination of other defects (e.g., ‘V_Mo_+I_C_’, ‘S_V-Mo_+V_C_’). The data with increasing S_V-Mo_ in (Mo, V)_2_C system is developed, and typical data (e.g., formation energy) for Mo-rich carbides and V carbides are correlated and the potential of the data in analysing transition of different carbides is highlighted. The relevance of using first principles calculation data in the studying of V-doping and the complex carbides (V- and Mo-rich carbides) evolution in different materials systems and future focus of continuous work is also discussed.

## 1. Introduction

Transition metal carbides (TMCs) are an important carbides group with high melting point and hardness, extremely high thermal and mechanical stability and excellent corrosion resistance to chemical reagents at room temperature [1,2,3]. In addition, TMCs have electrical and magnetic properties similar to their parent metals [4,5], which mean they are widely used in the field of cutting tools, mineral mining and anti-wear coatings, as well as nuclear reactors and catalysts [6,7,8,9]. Molybdenum carbide Mo_2_C is one key TCM carbides with promising applications in engineering and energy industries. Apart from the properties commonly shared by TMCs, Mo_2_C also has the electronic structure and catalytic functions similar to those of the precious metal Pt, which is opening up new prospects in a wide range of important applications in 2D materials, composite materials, energy storage, superconductivity and catalysis [10,11,12]. As a new functional material, Mo_2_C has the potential to replace the industrial hydrogenation catalyst with a very efficient catalytic effect, which has high importance to scientific research and technological developments [13,14,15,16,17].

Mo_2_C has been reported to have a variety of structures with different naming methods [18,19,20]. Among them, the two most commonly and widely reported Mo_2_C are α-Mo_2_C and β-Mo_2_C with orthorhombic and hexagonal close-packed structures, respectively. The majority of published work has been focused on fabrication, experimental structure analysis and property testing, as well as the application of Mo_2_C [21,22,23,24]. The stability of the structure, mechanical/thermal/electrical/magnetic properties of bulk material, surface adsorption and interface properties were studied through the first principles calculations of perfect defects-free Mo_2_C compounds. Liu et al. [25] studied the stability of α-Mo_2_C and β-Mo_2_C and found that α-Mo_2_C with orthorhombic structure is more stable than β-Mo_2_C with hexagonal structure according to the formation energy. In addition, the stability of both α-Mo_2_C and β-Mo_2_C increases by doping one Cr atom and decreases by doping one Nb atom. Wang et al. [26] calculated the melting point and hardness for both α-Mo_2_C and β-Mo_2_C based on density functional theory. Gibbs free energy for three types of Mo2C (o-Mo_2_C, h-Mo_2_C and t-Mo_2_C), as well as shear modulus and Young’s modulus were analysed in detail by Liu et al. [27]. Karaca et al. [28] estimated the value of the superconducting temperature of orthorhombic Mo_2_C based on the first principles calculation of the electron–phonon interaction. Yang et al. [29] calculated the surface energy of α-Mo_2_C(023) plane and found that the Y doping can facilitate the electron escaping from the α-Mo_2_C(023) surface, resulting in stronger surface activities. The work by Hassan et al. [30] revealed that various poisonous gas molecules can be chemisorbed on various locations of molybdenum carbide monolayer with high adsorption energies. Leitner et al. [31] built an interface model between Fe and Mo_2_C with different planes and terminations, the results show that the elasticity of the Fe/Mo_2_C composite is dominated by the elastic properties of the two materials, while the bonding at the interface only has a minor impact.

Among all the publications related to Mo_2_C, detailed studies on different types of defects in Mo_2_C are limited, which is relevant to both engineering and energy applications. One effective approach is through first-principles calculations, which has been used in studying defects in TMCs, such as monocarbides, including TiC, ZrC and HfC [32,33,34,35]. In this work, supercell structure of Mo_2_C was used to investigate the effects of vacancy, substitution and interstitial defects at different lattice sites. Vanadium-related defects and complex in β-Mo_2_C is systematically studied. The correlation between the first principles calculations data and vanadium doping effect in β-Mo_2_C, the potential phase in Mo–V steel systems [36,37,38,39,40,41] and recent and future works on phase engineering based on first principle calculation [42,43,44,45,46,47,48,49,50] is discussed.

## 2. Calculation Details

Mo_2_C belongs to the hexagonal crystal system P-3m1 (Z = 1) with initial lattice constant of the base cell of a = b = 0.3073 nm and c = 0.4653 nm. There are two Mo atoms and one C atom in the unit cell, which occupy the 2d position (Mo site (0.33, 0.67, 0.25) and (0.67, 0.33, 0.75)) and 1b position (C site (0, 0, 0.5)), respectively. Its crystal structure is shown in Figure S1 of the Supplementary Information. In this work, all DFT calculations were performed using the Cambridge Sequential Total Energy Package (CASTEP). Six electrons (4d^5^5s^1^) for Mo, five electrons (3d^3^4s^2^) for V, and four electrons (2s^2^2p^2^) for C were taken into account as valence electrons. The exchange–correlation functional was described by the Perdew–Burke–Ernzerhof (PBE) generalized gradient approximation (GGA). PBE is one of the most popular and reliable density functionals especially for the simulations of solid materials, which is widely used in the first principles calculations for TMCs themselves and different defects in TMCs and other types of intermetallic compounds. The Broyden–Fletcher–Goldfarb–Shanno (BFGS) algorithm was applied in the relaxation process of models to optimize the structures. The energy, maximum force and maximum displacement were set as 5 × 10^−6^ eV/atom, 0.01 eV/Å and 5 × 10^−4^ Å for the convergence tolerances, respectively. Based on convergence tests, which are shown in Figure S2, the Monkhorst–Pack k-point samplings of 6 × 6 × 6 and the plane–wave cutoff energy of 380 eV were used for Mo_2_C unit cell.

The size of the supercell was determined by calculating the formation energy of the vacancy defect in the supercell. First, perfect Mo_2_C supercells with size of 1 × 1 × 1 (3 atoms), 1 × 1 × 2 (6 atoms), 1 × 2 × 2 (12 atoms), 2 × 2 × 2 (24 atoms), 3 × 2 × 2 (36 atoms), 3 × 3 × 2 (54 atoms) and 3 × 3 × 3 (81 atoms) were built and optimized. Then the structures with one Mo vacancy (V_Mo_) or one C vacancy (V_C_) in different supercells were built and optimized. From Figure S3, as the size of the supercell increases, the formation energy of vacancy and bulk materials with vacancy gradually converge. So, 24-atom supercell with 2 × 2 × 2 replica of Mo_2_C unit cell was chosen to do the calculation. A similar supercell approach has been applied to other carbide systems (e.g., defect model in VC [41]). The domain of the super cell used and accuracy is comparable to other published works in similar systems [26,27].

Two types of the formation energy were considered in this paper: formation energy for intrinsic defects and formation energy for bulk material with defects, which are used to establish the stability of the structures with different point defects in Mo_2_C. Formation energy for point defects can be expressed as follows:

(1) $$E^{\mathrm{def}}=E(N)-E_{\mathrm{perf}}(N)+E(A)-E(B)$$

where $E^{\mathrm{def}}$ is formation energy for intrinsic defects; E(N) is the relaxed total energy of supercell with different point defects; $E_{\mathrm{per}}\left(N\right)$ is the relaxed total energy of the corresponding ideal supercell crystal; ‘A’ is the replaced or deleted atom and ‘B’ is the added atom compared to the ideal supercell; E(A) (or E(B)) is the energy per A(B) atom in pure A(B) crystal.

The formation energy for bulk material with defects is presented as follows:

(2) $$E^{\mathrm{bulk}}=\frac{E\left(N\right)\;-\sum_{i=1}^{3}n_{i}E_{i}}{\sum_{i=1}^{3}n_{i}}$$

where E^bulk^ is formation energy for bulk material with defects; E*_i_* is the energy per *i* atom in pure *i* crystal; *n* is the number of *i* atom in the supercell with defects.

Defect complexes, which contain two different types of defects, were also considered in this paper. The following equation describes the defect–defect interaction:

(3) $$E_{\mathrm{defect}-\mathrm{defect}}=E\left(N,\mathrm{defect}1\right)+E\left(N,\mathrm{defect}2\right)-\;E\left(N,2\mathrm{defects}\right)-{\;E}_{\mathrm{per}}\left(N\right)$$

where E(N,defect1) and E(N,defect2) are the relaxed total energy of supercell containing one defect in the system; E(N,2defects) is the relaxed total energy of supercell including two defects. The results calculated by Equation (3) are called binding energy between two defects.

## 3. Results

**Figure 1:**
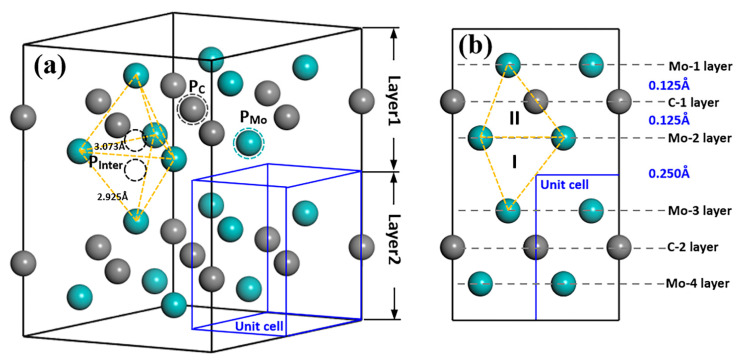
Schematic diagram of Mo2C structure of 2 × 2 × 2 supercell (**a**) and along [2$\bar{1}\bar{1}$0] direction (**b**). Cyan and grey balls represent Mo and C atoms; cyan and grey dashed circles are the positions that are used to form vacancy and substitution defects; yellow dashed frames are the tetrahedrons that Mo atoms formed inside the layer (type II) and between two layers (type I) and the black dashed circles are the tetrahedral position inside the tetrahedrons; blue frame refers to the Mo2C unit cell. PMo and PC represent the positions of the Mo and C atoms, while PInter refers to the position for interstitial atoms in the structure.

The model of intrinsic point defects that may exist in TMCs normally contains vacancies, substitutions (antisite) and interstitials. Interstitial defect includes octahedral interstitial and tetrahedral interstitial defects depending on the specific structure [32,33,34,35]. Figure 1 shows the models to investigate the effect of all different point defects on Mo_2_C crystal structure, including models with one atom vacancy (Mo vacancy (V_Mo_) and C vacancy (V_C_)), and one atom substitution (Mo substitution (S_Mo_) and C substitution (S_C_)), as well as one atom tetrahedral interstitials (Mo interstitial (I_Mo_) and C interstitial (I_C_)). From Figure 1a, V_Mo_ or V_C_ can be formed if P_Mo_ or P_C_ atom was deleted; moreover, the replacement of P_C_ atom by a Mo atom or P_Mo_ atom by a C atom will result in the formation of S_Mo_ or S_C_ defect. Here, P_Mo_ and P_C_ represent the positions of the Mo and C atoms. From Figure 1b, Mo_2_C supercell exhibits a clear layered structure. The distance between Mo and C atomic layers is 0.125 Å inside the layer, whereas the nearest distance between two Mo atomic layers from different layers is 0.250 Å. There are two tetrahedral interstitial positions for interstitial atoms: one is inside the same layer formed by four Mo atoms (type II) and the other one is between two layers (type I). When one Mo or C atom occupies P_Inter_ position, interstitial structures I_Mo_(I, II) or I_C_(I, II) can be obtained.

Vanadium-related defects in Mo_2_C supercell contain two different types, one is V substitutions and the other is V interstitials. V substitutions refer to the defects, for which, one V atom occupies P_Mo_ or P_C_ atom in the structure, as S_V-Mo_ and S_V-C_. Vanadium interstitial defects I_V_(I, II) are similar to I_Mo_(I, II) and I_C_(I, II), which means one Vanadium atom enters into the tetrahedral interstitial positions P_Inter_ inside the layer (type II) or between two layers (type I).

### 3.1. Intrinsic Defects

#### 3.1.1. Formation Energy and Stability

As mentioned in Section 2 that the size of supercell was determined by calculating the formation energy of the vacancy defect for Mo_2_C structure of different sizes. Table S1 lists the total energy for different Mo_2_C supercells with or without vacancy, from which formation energy for vacancy defects in Table S2 can be calculated. It can be seen that all calculated total energies for different supercells are negative. As the number of atoms of the supercell increases, the absolute value of total energy increases as well. For the same supercell, the absolute total energy value of the supercell containing a vacancy is smaller than that for the perfect structure. In addition, the absolute value of the total energy for the supercell with V_C_ is larger than that with V_Mo_.

Tables S3 and S4 lists the total energy of Mo_2_C supercells with substitution, interstitial and V-related defects. When an atom with a higher atomic number replaces an existing atom with a lower atomic number, the absolute total energy of supercell rises, and *vice versa*. If an additional atom was added in the supercell forming interstitial defects and the absolute total energy is increased. Moreover, note that the values of total energy for supercells with I_Mo_ or I_C_ are exactly the same although the Mo or C atom occupies different interstitial positions I and II, which cause the same formation energy of I_Mo_(I) and I_Mo_(II), as well as I_C_(I) and I_C_(II) from Table 1.

According to Equations (1) and (2), the formation energy of point defects and bulk materials with different defects was calculated and presented in Table 1. All formation energy values of the point defects are positive, which suggests that all the point defects are not more energetically favourable compared to perfect Mo_2_C. Among all the intrinsic defects, the formation energy for V_C_ defect is the lowest, which shows that V_C_ is the most stable defect and its chance to exist in the crystal is the highest, followed by I_C_(I,II) and S_C_. It also can be seen that C-related defects are more favourable than metal-based defects, which is similar to the case in other transition–metal carbides, such as VC and TiC [41].

Table 1 also lists the formation energy of Vanadium-related defects in Mo_2_C crystal. It is very interesting to note that S_V-Mo_ is the only defect that has a negative formation energy value of point defects and bulk materials with defect, which means that this defect has better stability and it is a favourable trend for Mo atoms to be replaced by V atoms in the crystal.

#### 3.1.2. Structures and Electronic Properties

##### V_C_ and S_V-Mo_

**Figure 2:**
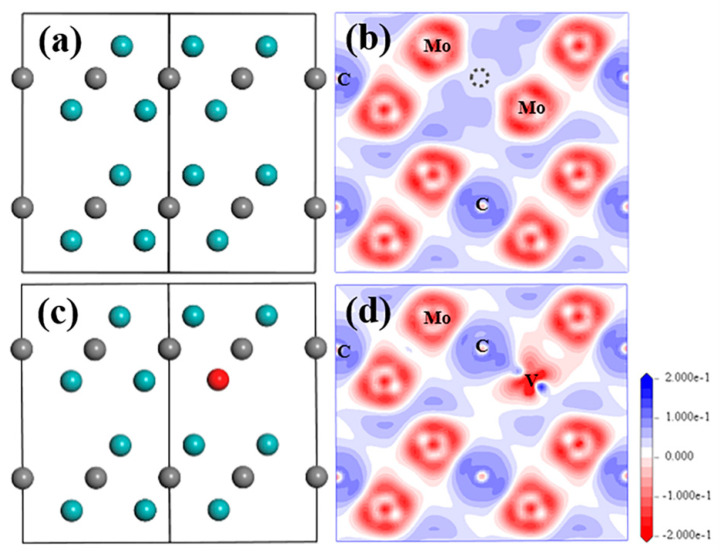
Structure model and electron density difference distribution (EDDD) after optimization in ($\bar{1}\bar{1}$20) plane of Mo_2_C supercell with V-related defect of (**a**,**b**) V_C_ and (**c**,**d**) S_V-Mo_. Cyan and grey balls represent Mo and C atom, red ball represent V atom; grey dashed circle shows the V_C_ position.

Based on the modelling work of the structure analysis and optimisation, it is found that S_V-Mo_ and V_C_ are the two most stable defects among all point defects. The negative formation energy S_V-Mo_ strongly suggests that it will exist in the structure. Figure 2 shows the optimized structures with these two defects and their electron density difference distribution (EDDD) in [11$\bar{2}$0] plane, and the Mulliken atomic charge of Mo and C atoms in all relaxed structures, as well as bond population for Mo–C bond are listed in Table 2. For the perfect Mo_2_C structure, all Mo atoms have the same charge number 0.3, and all C atoms have the same charge number −0.6. After a point defect was introduced, there is more than one charge number for the same species, especially for the metal element Mo because the electron distribution is not symmetric anymore. The charge varies significantly, especially for the atoms, which are very close to the defect or the defect atom itself, such as the substituted Mo atom in S_Mo_ with the charge number of −0.1, as well as the substituted C atom in S_C_ with the charge number of −0.37, both of which show big differences from their original charge.

From Figure 2a, nearly all atom positions for the V_C_ situation seem to be unchanged. The existence of V_C_ did not change the EDDD result (Figure 2b) and the atomic charge of the Mo and C atom (Table 2) significantly compared to data for the perfect structure. The strongest bonds are formed between C atoms in the C-1 layer and Mo12 in the Mo-1 layer, as well as Mo13 in Mo-2 layer (Figure S4) with the bond population of 0.41. From the analysis of the electronic property of Mo_2_C with V_C_, it can be noted that the atoms in Layer1 barely interact with the atoms in Layer2, maybe because of the larger distance between these two layers, which also reflects the lamellar structure of this compound.

When S_V-Mo_ defect exists in the structure, it can be seen from Figure 2c that sub-V atom can fit perfectly in the original Mo position without obvious replacement after relaxation. Apart from the sub-V atom, all Mo and C atoms in this supercell exhibit a negligible displacement, eventually forming a perfect stable crystal structure. Similar situation in respect of electronic properties was observed for S_V-Mo_ as well. No obvious change of electron transfer was observed for all Mo and C atoms in EDDD result, as shown in Figure 2d, while more electron loss is only shown in the surrounding area of the sub-V atom compared to the Mo atoms in the structure. This finding is also consistent with the atomic charge of Mo, C and sub-V atoms in Table 2. The atomic charge values of the Mo atom and C atom, as well as the Mo–C bond population in the supercell are comparable to those for perfect structure and structure with V_C_ defect. Sub-V atom is found to lose more electrons due to higher atomic charge 0.71e, but the bond population of V–C is smaller than all Mo–C bonds with a value of 0.23.

##### S_C_ and I_C_

**Figure 3:**
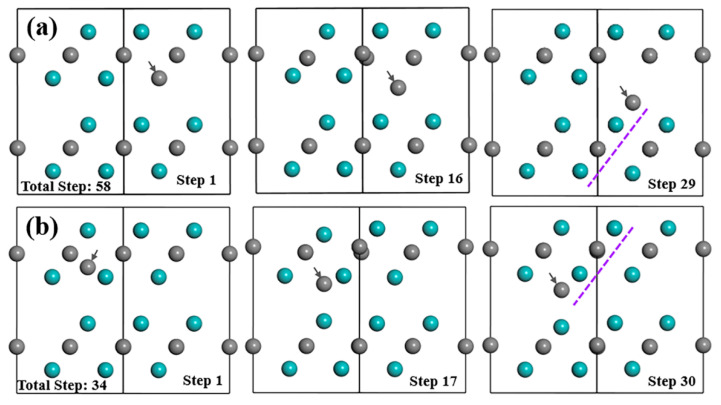
Structure evolution of Mo_2_C supercell with (**a**) S_C_ and (**b**) I_C_(II) during optimization process. Cyan and grey balls represent Mo and C atoms, respectively; grey arrows refer to the defect atom.

**Figure 4:**
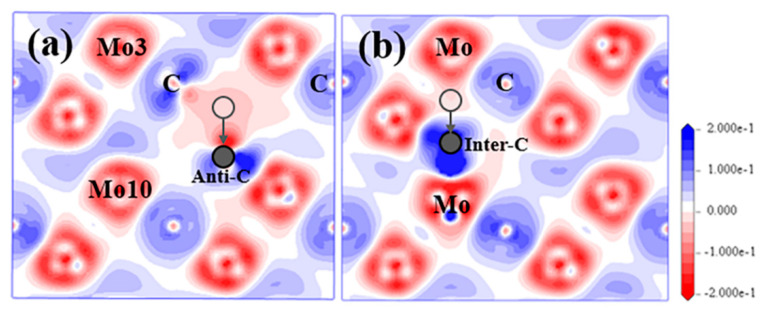
EDDD for Mo_2_C supercell with (**a**) S_C_ and (**b**) I_C_(II) in ($\bar{1}\bar{1}$20) plane. Semitransparent grey balls represent the original position for C atom; Solid grey balls refer to the final position for defect C atom.

From Figure 3a, if the substitutional C atom exists in the Mo-2 layer, most atoms in this structure will not move within their own planes, as well as along the [1] direction. However, during the optimization process, the anti-C atom will move from its original place downwards gradually to the place that is very close to Layer2 and form the strongest bond with the Mo atoms in the Mo-3 layer with the bond population of 0.63 from Table 2 and Figure S5. Anti-C atom finishes moving at step-29 out of the total steps of 58 of the optimization process, and finally stabilised close to the type I interstitial position of the tetrahedron. This process tends to extend the lattice structure of Layer2 as indicated by the purple dashed lines in Figure 3a. After relaxation, the atomic charge for the Mo and C atom and the bond features are more complex than the above-discussed V_C_ and S_V-Mo_ situation. The electrons that the anti-C atom gained are much less (−0.37e) than normal C atoms (−0.6e). The change of atomic charges for both the Mo3 atom in Layer1 in Figure 4 and the Mo10 atom at the same place in Layer2 is the largest. The Mo3 atom loses the most electrons (0.43e), while the Mo10 atom lose the least electrons (0.16e) among all Mo atoms. In addition, it is interesting to note that the Mo_2_C supercell with S_C_ transformed into a structure similar to defect complex V_Mo_+I_C_(I). Mo_2_C supercell with defect complex V_Mo_+I_C_(I) was also built and analysed in detail; the results are shown in Section 3.2.1.

After an interstitial C atom was added in the tetrahedron surrounded by Mo atoms within the same layer to form type II interstitial defects, inter-C atom cannot stay stably in the type II interstitial position during the relaxation process, which is shown in Figure 3b. Instead, it will move downwards gradually, go through Mo-2 layer and finally occupy the type I interstitial position between the two layers. Similar to situation for S_C_, inter-C also tends to arrange in the Layer1 lattice and extend the lattice structure. After structure optimization, inter-C(II) atom and inter-C(I) atom have similar atomic coordinates and the atom distributions in both structures are almost the same. I_C_(I) and I_C_(II) also show comparable electronic properties after relaxation. The inter-C atom obtains a few more electrons than the original C atoms in the structure with the atomic charge of −0.58e, and the bonds between the inter-C atom and Mo atoms at the vertices of the tetrahedron are the strongest among all Mo–C bonds in the supercell. These Mo atoms lose the most electrons with the largest atomic charge of 0.44e.

The cases of I_C_(I) and I_C_(II) can be applied for Mo_2_C supercell with Mo and V interstitial defect as well (Figure S6). Inter-V/Mo(I) atom will shift near to its original position, while inter-V/Mo(II) atom will move downwards to the position close to the interstitial position II between the two layers. Structures of I_Mo_(I) and I_Mo_(II) have the same electronic properties after relaxation, which can be seen from Table 2, while I_V_(I) and I_V_(II) exhibits a slight difference from each other.

### 3.2. Defect Complexes

There are so many combinations of defect complexes if all intrinsic defects are considered. In this section, three types of defect complexes were chosen and analysed designated as: (1) V_Mo_+I_C_, (2) S_V-Mo_+V_C_ and (3) S_V-Mo_+S_V-Mo_. It is already mentioned in Section 3.1.1 that the S_C_ defect tends to transform into V_Mo_+I_C_(I) after relaxation, so V_Mo_+I_C_(I) may be stable as a preferable type of defect in the structure. From the calculated formation energy for all intrinsic defects, S_V-Mo_ and V_C_ are the two main stable defects that are most likely to exist in the Mo_2_C supercell, so the defect combination of S_V-Mo_ and V_C_ is considered in this part. In addition, S_V-Mo_ is the defect with the strong negative formation energy of the defect, so maybe more V atoms tend to replace Mo in the structure, so S_V-Mo_+S_V-Mo_ and more combinations will be considered in this section as well.

#### 3.2.1. V_Mo_+I_C_(I)

**Figure 5:**
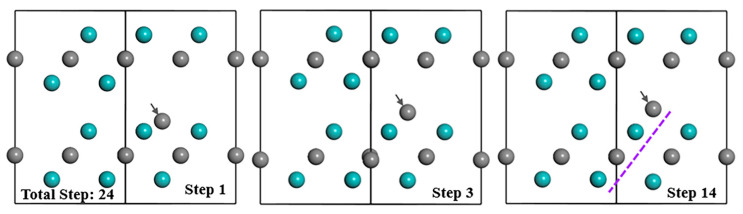
Structure evolution of Mo_2_C supercell with V_Mo_+I_C_(I) during optimization process. Cyan and grey balls represent the Mo and C atoms respectively; grey arrows refer to the defect atom.

During the relaxation process of the Mo_2_C supercell with V_Mo_+I_C_(I) defect complex, inter-C atom moves upward and reaches the final position at step 3 (Figure 5). After step 3, inter-C atom barely moves, but other atoms continue to vibrate until step 14. Eventually, inter-C atom arranges in the lattice Layer2 extending the structure and other atoms still occupy the original positions, which are similar to the situation of S_C_ and I_C_. Additionally, the atom distribution, electron distribution and atomic charge of Mo and C atoms, as well as the bond population of the Mo_2_C supercell with V_Mo_+I_C_(I) are nearly the same as that with S_C_ if the values from Table 3 are compared with Table 2. Even their formation energy values of point defects and bulk materials with defect from Table 4 are almost the same after optimization, which means that V_Mo_+I_C_(I) and S_C_ are exactly equivalent and V_Mo_+I_C_(I) is not a clear favourable defect (with better stability) that can exist in the structure.

It is known that when two defects exist in the structure at the same time, the binding energy can be calculated according to Equation (3). If the binding energy is negative, these two defects repel each other, whereas a positive value of binding energy indicates that the two defects attract each other. From Table 4, the positive value of binding energy between V_Mo_ and I_C_(I) manifests a certain level of attraction between them leading to the obvious movement of the inter-V atom towards the direction of Mo vacancy.

#### 3.2.2. S_V-Mo_+V_C_

**Figure 6:**
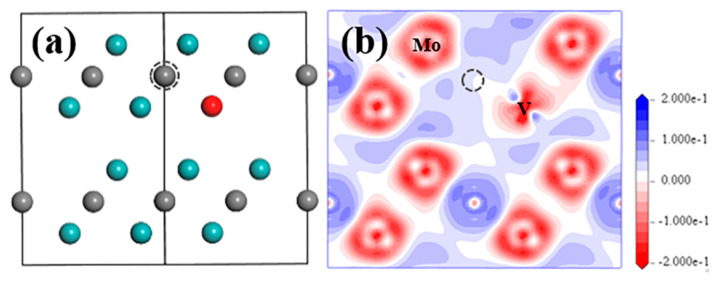
Structure model (**a**) and EDDD (**b**) after optimization in ($\bar{1}\bar{1}$20) plane of Mo_2_C supercell with S_V-Mo_+V_C_ defect. Cyan and grey balls represent Mo and C atom, red ball represents V atom; grey dashed circle shows the V_C_ position.

When S_V-Mo_ and V_C_ defects exist at the same time, there is no obvious change for the positions of all atoms in the structure (Figure 6a). After simulation, the atom positions seen from [$\bar{1}\bar{1}$20] direction are almost the same as with those for S_V-Mo_ in Figure 2c. Moreover, limited changes have been identified in the electronic properties of S_V-Mo_+V_C_ compared to those for S_V-Mo_, such as EDDD from Figure 6a, atomic charge and bond population (Table 3).

From Table 4, S_V-Mo_+V_C_ has relatively small formation energy values of point defect and for bulk material, slightly higher than the data for of S_V-Mo_ and V_C_. This suggests that the chance of existence of this defect complex is quite a lot higher than other intrinsic defects or V-related defects. However, due to the positive value of the formation energy of point defect, this defect complex is not the must-existed one. The binding energy for S_V-Mo_+V_C_ is negative, and the absolute value is almost 50% lower than that for V_Mo_+I_C_, which indicates that S_V-Mo_ and V_C_ tend to repel each other but not significantly strongly.

#### 3.2.3. S_V-Mo_ Defect Combinations

**Figure 7:**
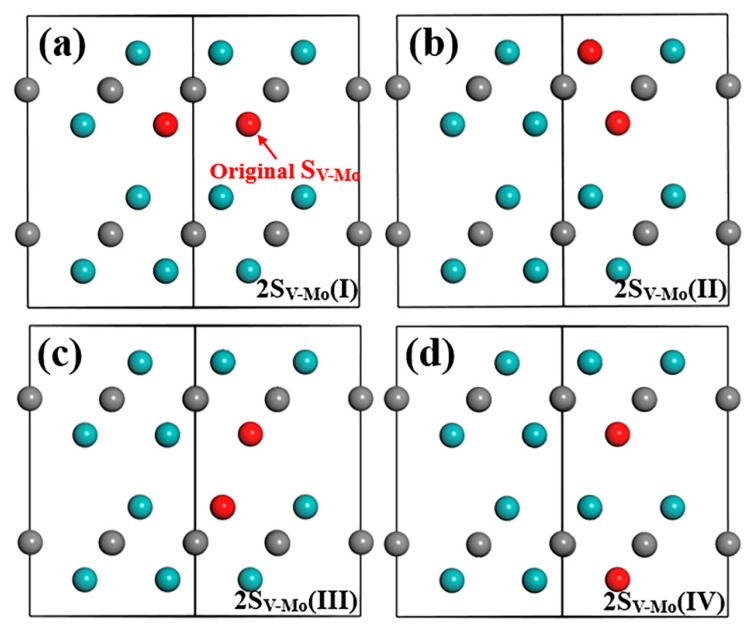
Structure model after optimization in ($\bar{1}\bar{1}$20) plane of Mo_2_C supercell with two S_V-Mo_ defects: (**a**) original S_V-Mo_+ S_V-Mo_ in Mo-2 layer (same layer), (**b**) original S_V-Mo_+ S_V-Mo_ in Mo-1 layer (different layer in Layer1), (**c**) original S_V-Mo_+ S_V-Mo_ in Mo-3 layer (different layer in Layer2) and (**d**) original S_V-Mo_+ S_V-Mo_ in Mo-4 layer (different layer in Layer2). Cyan and grey balls represent Mo and C atom, red ball represents V atom.

In this section, four types of S_V-Mo_+S_V-Mo_ combination are first considered. The original S_V-Mo_ defect is indicated by a red arrow in Figure 7. Due to different Mo positions in the supercell, the combination structures between the original S_V-Mo_ (discussed above) and other S_V-Mo_ defects in the original layer or in different Mo layers in Layer1 and Layer2 are established and calculated. After structure optimization, all atoms stay in the same position for these four models. The total energy of these models is listed in Table 5 and all of them have almost the same energy values as represented by the same formation energy for defect and bulk material. This finding implies that two V atoms can randomly occupy any original Mo lattice coordinate without obvious bias. As long as one S_V-Mo_ defect forms, a new V atom may appear at any Mo position and replace the Mo atom. In addition, the formation energies of the defect and bulk material are both negative and the values are low compared to all other single defects and defect complexes. Note that among all the defects we discussed above, S_V-Mo_ has the smallest formation energy value, suggesting that it is the most stable defect, which will exist in the structure. When two S_V-Mo_ exist in Mo_2_C supercell, it is found (Table 5) that the formation energy values are even lower than single S_V-Mo_ defect, which means that the S_V-Mo_+S_V-Mo_ combination is more stable than single S_V-Mo_.

## 4. Discussion

Defects in crystal and its influence on energy and structures are important for crystal formation, evolution and phase transitions. The data selected in papers show that first principles calculation is an effective way to study the effect of the different defects and establish some important data and trends. The systematic data obtained with both positive (with clear effect) and negative (no major effects) are all relevant to some important issues, which cannot be directly obtained solely/effectively from experimental means. Similar to other transition metal carbides (TMCs), such as monocarbide VC, TiC, and NbC, Mo_2_C tends to have C-related defects in the structure. The data show that V_C_ is the most stable defect among all vacancy, substitution and interstitial defects. However, different from V_C_ defects in other TMCs (such as TiC, VC, NbC) [41,42,43] for which will Vc exist inevitably, V_C_ in Mo_2_C just has the possibility to appear in the structure due to the positive value of the formation energy of point defects (Table 1). In addition, interstitial sites II inside the same layer (Layer1 or Layer2) shown in Figure 1 are not a preferable or stable position for any type of atoms (Mo, V and C). The detailed analysis of the geometry optimisation data shows that the atoms always move to the interface area between the two layers. The typical case is I_C_(II), as evident in Figure 3b. When there are C atoms existing between the layer interface, they tend to arrange next to the Mo atom and expand the lattice structure, which can be seen from examples, such as S_C_, I_C_(I) and V_Mo_+I_C_(I), in Figure 3a and Figure 5.

**Figure 8:**
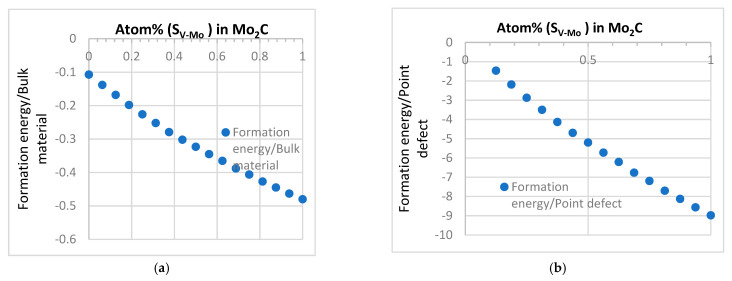
The bulk formation energy and the formation energy of defect with different S_V-Mo_ concentration. (**a**) Formation energy. (**b**) Formation energy of defect.

The data suggest that V-related defect, S_V-Mo_, is the one that has a clear negative formation energy value as the lowest case among all single point defects. This is different from the effect of the Mo atom in vanadium carbide (VC), as shown in a previous work by the authors [41]. It was found that VC is a very stable structure, and the only defect that can exist stably in VC is the C vacancy. Even though Mo has the tendency to enter the lattice structure and replace the V atom (S_Mo-V_), the existence of Mo in VC is still not stable based on the energy data [41]. However, the data established in this work clearly show that when a V atom replaces Mo in β-Mo_2_C structure, S_V-Mo_ is the main stable point defect with a low formation energy (Table 1). It is also interesting to note from the systematic data for defect complexes, S_V-Mo_ is the most stable defect in Mo_2_C but it has a rather limited effect on stabilizing carbon vacancy V_C_ and vice versa. This suggests that the combination of S_V-Mo_ and V_C_ cannot make the structure more stable compared to the influence of the single type of defects of S_V-Mo_ and V_C_. The data indicate that a potential way to improve the stability of the structure is to increase the concentration of S_V-Mo_. The result of the formation energy values in Table 1 and Table 5 imply a trend that as more V atoms replace Mo atoms, the structure may become more stable. Systematic supercell models have been developed with increasing number of V atoms replacing Mo in Mo_2_C; some typical data are shown in Figure 8.

As shown in Figure 8, respectively, the bulk formation energy and the formation of the defects decreased with the number of S_V-Mo_. The data for pure Mo_2_C and V_2_C are compared against data published calculations and experimental data [25,26,46,47]. The data show that the formation of energy of (Mo, V)_2_C decreased smoothly with increasing number of V atoms replacing Mo in the system. This reflects the structure similarity between these two types of compounds. These are relevant to understanding the doping effect. In addition, given the wide application of Mo and V in steels and exists of different types of carbides [36,37,38,44,45,48], the data obtained of this nature may provide useful information/direction on phase formation/transition in steel/alloy design as increasingly the first principles calculations data are used in predicting the possible phase transitions in different systems [45,48,49]. The trend between energy and the concentration of S_V-Mo_ is similar to the observation of another published work on applying first-principle study on the stability of lightly doped (Nb1−xTix)C complex carbides and their verification in 1045 steel [49]. In the work, the effect of Ti atoms sequentially replacing zero, three, six, and nine Nb atoms in nonstoichiometric carbides (Nb1−xTix)C is studied, the total energy of (Nb1−xTix)C is found to be correlated to the number of Ti atoms following a general linear trend. As an important development in terms of the effective use of the first principles approaches in alloys [46], the data/trend established were successfully used to guide the design of the complex carbides and correlated to purposely designed experimental data. This is an effective application case of combining first principles calculations and alloy design for phase engineering. The Mo–V system is drawing increasing attention recently. Mo_2_C is an important material relevant to many different functions including mechanical, catalyst and, more recently, H_2_ trapping, including V-doped Mo_2_C [50,51,52,53,54]; the data established of the structural and energy characteristics and the effect of different defects and defect complexes in Mo_2_C is relevant to many focuses of continuous and further studies. One area is the data on the magnetic moments, electrical and other functional properties. Future work will systematically screen the effects of defects in the magnetic moment in Mo_2_C in comparison with other simple or multicomponent carbides. The work on defects is also relevant to the effect of radiations. Even though the materials studied are not widely used as a bulk ceramic in nuclear-related applications, but they are related to some advanced alloys that serve under the radiation environment of different scales. The sensitivity of different defects and materials to radiation is of interest for future studies. It is easy to link the work to the direct doping of V of Mo_2_C [50,51]. For example, increasing the V content in β-(Mo_1–x_V_x_)_2_C retards the onset of reduction and strongly influences the kinetics of carburization [50]. In the work by Cotter et al. [52], (Mo_x_, V_1−x_)_2_C was found to have a high surface area. The Mo–V carbide system in alloys is important but more complicated. β-Mo_2_C has more a complex structure than other cubic carbides system, the data related to Mo_2_C and its interaction with other elements need further systematic studies. In steels containing Mo, with no nitrogen, M2X is often close to the ideal Mo_2_C composition with HCP structures (β) [53]. In previous works [36,37,38,39,40], M_2_C was identified as a Mo-rich carbide in Fe–Cr–W–Mo–V–C alloys, and there is a tendency for Mo_2_C disappearing gradually and transforming into V-dominated carbide eventually [40]. A recent work by Seo et al. [53] show that V–Mo system could effectively increase its hydrogen-trapping capability but clarifying the precipitation of Mo carbides need to be further investigated. The favourable trend of V replacing Mo could potentially offer additional insight/data to the understanding of Mo-related carbides, which is difficult to be observed in full experimentally. This will help unlock some key links of knowledge in phase formation and transformation in advanced alloy design by combing the framework of first principles calculations (detailed data/trend analysis) and thermal dynamic simulations with controlled advanced experiments [39,40,49,53,55,56,57,58,59,60].

## 5. Conclusions

This work developed systematic data using first principles calculations of energy and structure of the point defects, Vanadium-related defects and defect combinations (vacancy (V), substitutional (S) and/or interstitial (I)) in molybdenum β-Mo_2_C.) Detailed analysis shows that C-related defects are more favourable than metal-based defects and V_C_ is the most stable defect among all the single intrinsic defects in pure β-Mo_2_C. However, due to having a higher formation energy than the perfect structure, V_C_ is not the inevitably existed defect. S_V-Mo_ has negative and the smallest formation energy value making it the most stable defect among all single point defects and V-related defects. The data show that interstitial site II is a very unstable position for any type of atoms (Mo, V and C) in β-Mo_2_C, and the atom always moves to the interface area near interstitial site I between two layers. The C atom tends to arrange next to the Mo atom and extend lattice structure if it exists between the layer interfaces. The formation energy of the bulk material and the formation energy of the defects continuously decreases with an increasing number of S_V-Mo_. The data with increasing S_V-Mo_ in the (Mo, V)_2_C system are developed, and typical data (e.g., formation energy) for Mo-rich carbides and V carbides are correlated, and the potential use of the data in analysing the transition of different carbides is highlighted. The use of first principles calculation data for analysing the link between different Mo–V carbides and the effects of doping elements is discussed. The relevance of using first principles calculation data in studying complex carbides evolution and future focus is also highlighted.

## Figures and Tables

**Table 1:** Formation energy (eV) of point defects and bulk material with defects for Mo_2_C.

Supercell	Formation Energy	
	Point Defect	Bulk Material
Perfect		−0.051
V_Mo_	3.833	0.071
V_C_	0.410	−0.039
S_Mo_	7.487	0.205
S_C_	4.232	0.070
I_Mo_(I,II)	10.252	0.308
I_C_(I,II)	3.210	0.026
S_V-Mo_	−0.761	−0.138
S_V-C_	5.623	0.128
I_V_(I)	7.820	0.211
I_V_(II)	6.991	0.177

**Table 2:** Mulliken atomic charge and bond population in the supercell with different defects. In perfect Mo_2_C structure, atomic charge for Mo and C atom is 0.3 and −0.6, respectively, and all Mo–C bond populations are 0.33. Note: the numbers in the table may vary by ±0.2.

Defect	Atomic Charge (e)			Bond Population	
	Mo	C	V	Mo–C Bond	V–C Bond
V_Mo_	0.36, 0.21, 0.07	−0.61, −0.54	—	0.51, 0.42, 0.35, 0.30, 0.27, 0.13	—
V_C_	0.28, 0.26	−0.61	—	0.41, 0.33, 0.28	—
S_Mo_	0.31, 0.22, −0.1	−0.61, −0.58	—	0.43, 0.33	—
S_C_	0.43, 0.34, 0.16	−0.62, −0.54, −0.37	—	0.63, 0.53, 0.42, 0.37, 0.32, 0.27, 0.13	—
I_Mo_(I)	0.54, 0.34, 0.28, 0.09, −0.02	−0.61	—	0.4, 0.36, 0.32, 0.23, 0.12	—
I_Mo_(II)	0.54, 0.34, 0.28, 0.09, −0.02	−0.61	—	0.4, 0.36, 0.32, 0.23, 0.12	—
I_C_(I)	0.44, 0.33, 0.29, 0.23	−0.61, −0.58	—	0.38, 0.36, 0.33, 0.29, 0.26	—
I_C_(II)	0.44, 0.33, 0.29, 0.23	−0.61, −0.58	—	0.38, 0.36, 0.33, 0.29, 0.26	—
S_V-Mo_	0.29, 0.26	−0.61	0.71	0.38, 0.32	0.23
S_V-C_	0.32, 0.26, 0.13	−0.6	0.33	0.36, 0.33	—
I_V_(I)	0.34, 0.31, 0.11, 0.05	−0.61	0.56	0.36, 0.31, 0.12	−0.04
I_V_(II)	0.33, 0.29, 0.11, 0.07	−0.6	0.52	0.36, 0.31, 0.15	0

**Table 3:** Mulliken atomic charge and bond population in the supercell with different defect complexes. In perfect Mo_2_C structure, atomic charge for Mo and C atom is 0.3 and −0.6, respectively, and all Mo–C bond populations are 0.33. Note: the numbers in the table may vary by ±0.2.

Defect	Atomic Charge (e)			Bond Population	
	Mo	C	V	Mo–C Bond	V–C Bond
V_Mo_+I_C_	0.43, 0.34, 0.15	−0.62, −0.54, −0.37	—	0.64, 0.53, 0.42, 0.37, 0.32, 0.27, 0.13	—
S_V-Mo_+V_C_	0.26, 0.23, 0.21	−0.61	0.64	0.45, 0.40, 0.33, 0.28	0.21

**Table 4:** Calculated formation energy and binding energy of Mo_2_C supercell with defect complexes (eV).

Supercell	Total Energy	Formation Energy		Binding Energy
		Point Defect	Bulk Material	
V_Mo_+I_C_	−30,446.788	4.307	0.073	1.635
S_V-Mo_+V_C_	−32,116.798	0.641	−0.083	−0.768

**Table 5:** Calculated formation energy and binding energy of Mo_2_C supercell with different S_V-Mo_ combinations (eV).

Supercell	Total Energy	Formation Energy		Binding Energy
		Point Defect	Bulk Material	
2S_V-Mo_(I)	−32,313.597	−1.464	−0.168	−0.058
2S_V-Mo_(II)	−32,313.602	−1.464	−0.168	−0.058
2S_V-Mo_(III)	−32,313.615	−1.464	−0.168	−0.058
2S_V-Mo_(IV)	−32,313.642	−1.464	−0.168	−0.058

## Supplementary Materials

The following supporting information can be downloaded at: https://www.mdpi.com/article/10.3390/ma15134719/s1.
