# Supplementary material for: Insight into Point Defects and Complex Defects in β-Mo2C and Carbide Evolution from First Principles
Source: Materials (Basel). 2022 Jul 5;15(13):4719. doi: 10.3390/ma15134719 (PMC9267726; doi:10.3390/ma15134719)
Supplement: Supplementary file 1 [file materials-15-04719-s001.zip › materials-1758422-Supplementary.pdf]

Supplementary information

Insight into point defects in β-Mo2C and carbide evolution from first principles

Jing Guo, Yunli Feng, Xiaoliang Qing, Cong Tang, Li Wang, Qingxiang Yang, Xuejun Ren

Figures

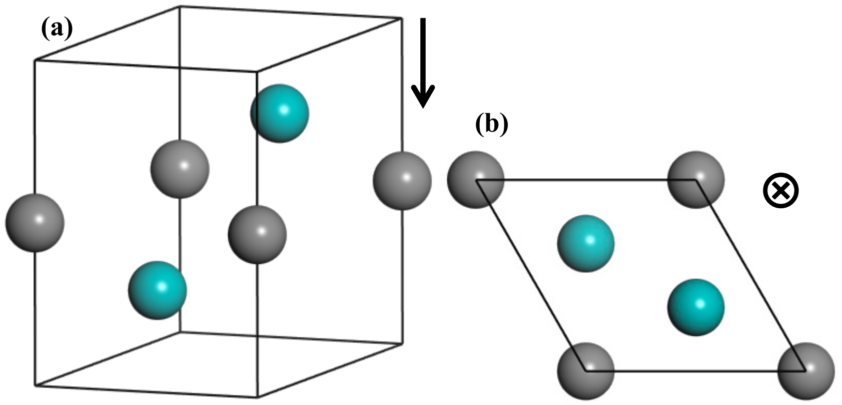

Figure S1 Schematic diagram of Mo2C structure of unit cell (a) and along (b) [0001] direction.

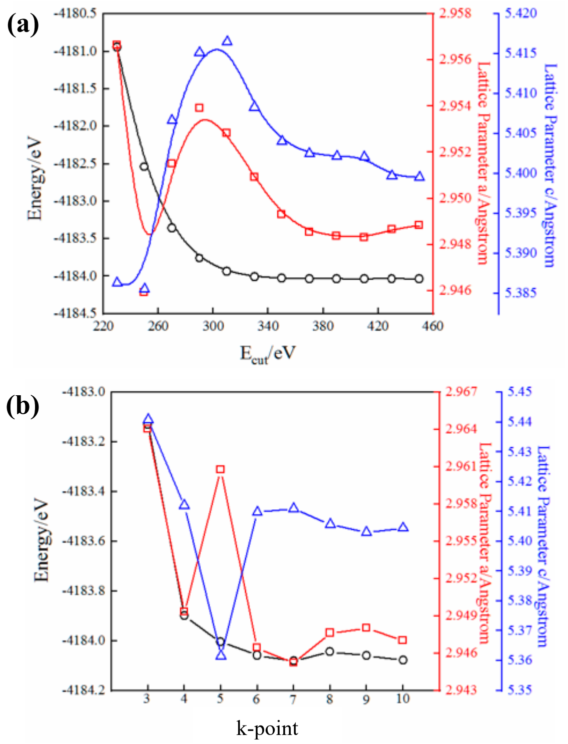

Figure S2 Convergence tests for Mo2C unit cell: Change of total energy and lattice parameter for Mo2C with different (a) cutoff energies and (b) k-points.

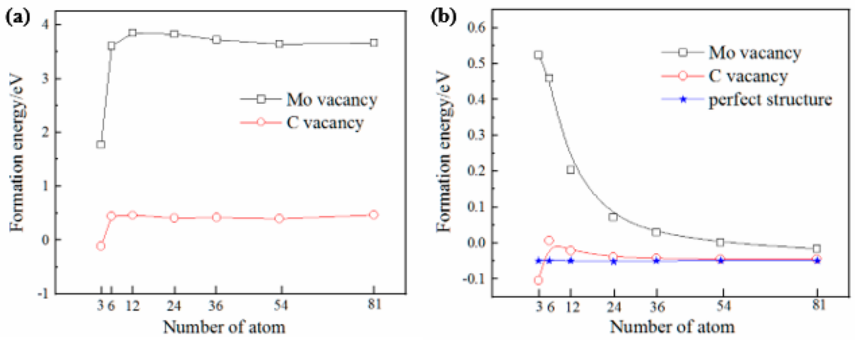

Figure S3 Convergence test for Mo2C supercell: (a) formation energy of vacancy, (b) formation energy of bulk material with vacancy.

(a)

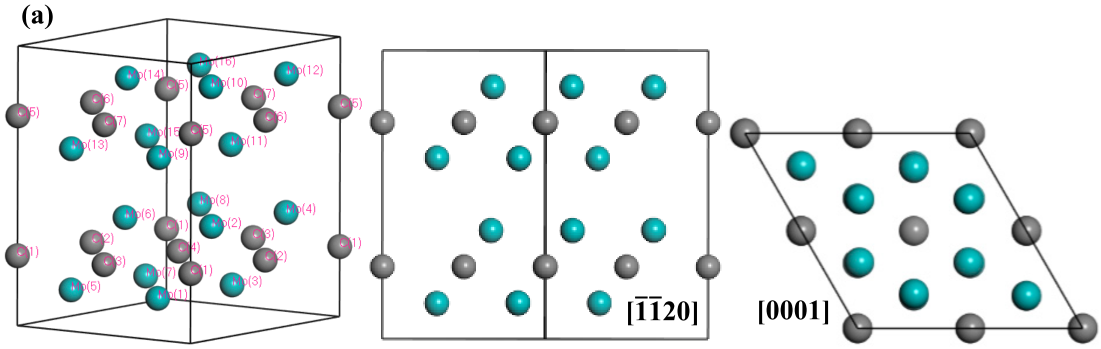

(b)

Mulliken atomic populations for V_C

Species	No.	s	p	d	f	Total	Charge(e)
C	1	1.44	3.17	0	0	4.6	-0.6
C	2	1.44	3.17	0	0	4.61	-0.61
C	3	1.44	3.17	0	0	4.61	-0.61
C	4	1.43	3.18	0	0	4.61	-0.61
C	5	1.44	3.18	0	0	4.62	-0.62
C	6	1.44	3.18	0	0	4.62	-0.62
C	7	1.44	3.18	0	0	4.62	-0.62
Mo	1	2.21	6.63	4.88	0	13.72	0.28
Mo	2	2.21	6.63	4.88	0	13.72	0.28
Mo	3	2.21	6.63	4.88	0	13.72	0.28
Mo	4	2.21	6.64	4.87	0	13.72	0.28
Mo	5	2.21	6.64	4.87	0	13.72	0.28
Mo	6	2.21	6.63	4.88	0	13.72	0.28
Mo	7	2.21	6.63	4.88	0	13.72	0.28
Mo	8	2.21	6.63	4.88	0	13.72	0.28
Mo	9	2.22	6.63	4.89	0	13.74	0.26
Mo	10	2.22	6.63	4.89	0	13.74	0.26
Mo	11	2.22	6.63	4.89	0	13.74	0.26
Mo	12	2.21	6.66	4.87	0	13.74	0.26
Mo	13	2.21	6.66	4.87	0	13.74	0.26
Mo	14	2.22	6.63	4.89	0	13.74	0.26
Mo	15	2.22	6.63	4.89	0	13.74	0.26
Mo	16	2.22	6.63	4.89	0	13.74	0.26

(c)

Bond populations for V_C

Bond	Population	Length(Å)	Bond	Population	Length(Å)
C 7 Mo 12	0.41	2.08871	C 3 Mo 1	0.33	2.10658
C 6 Mo 12	0.41	2.08871	C 3 Mo 4	0.34	2.11224
C 7 Mo 13	0.41	2.08871	C 2 Mo 4	0.34	2.11224
C 6 Mo 13	0.41	2.08871	C 3 Mo 5	0.34	2.11224
C 5 Mo 12	0.41	2.09045	C 2 Mo 5	0.34	2.11224
C 5 Mo 13	0.41	2.09046	C 1 Mo 4	0.33	2.11324
C 4 Mo 2	0.32	2.10123	C 1 Mo 5	0.33	2.11325
C 4 Mo 8	0.32	2.10123	C 5 Mo 16	0.28	2.12091
C 4 Mo 7	0.32	2.10123	C 5 Mo 10	0.28	2.12091
C 4 Mo 1	0.32	2.10123	C 5 Mo 9	0.28	2.12091
C 4 Mo 6	0.32	2.10288	C 5 Mo 15	0.28	2.12091
C 4 Mo 3	0.32	2.10289	C 6 Mo 14	0.28	2.12125
C 1 Mo 8	0.33	2.10557	C 7 Mo 14	0.28	2.12125
C 1 Mo 2	0.33	2.10557	C 6 Mo 11	0.28	2.12125
C 1 Mo 1	0.33	2.10557	C 7 Mo 11	0.28	2.12125
C 1 Mo 7	0.33	2.10557	C 7 Mo 16	0.28	2.12161
C 2 Mo 6	0.33	2.10578	C 6 Mo 10	0.28	2.12161
C 2 Mo 6	0.33	2.10578	C 7 Mo 9	0.28	2.12161
C 1 Mo 3	0.33	2.10578	C 6 Mo 15	0.28	2.12161
C 3 Mo 3	0.33	2.10578			
C 3 Mo 8	0.33	2.10657			
C 2 Mo 2	0.33	2.10657			
C 2 Mo 7	0.33	2.10658			

Figure S4 Structure model of Mo2C supercell with V_C after optimization (a), as well as atomic population (b) and bond population (c).

(a)

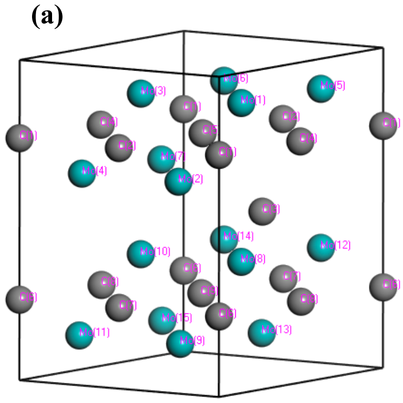

(b)

Mulliken atomic populations for S_C

Species	No.	s	p	d	f	Total	Charge(e)
C	1	1.45	3.18	0	0	4.63	-0.63
C	2	1.46	3.08	0	0	4.54	-0.54
C	3	1.55	2.83	0	0	4.37	-0.37
C	4	1.46	3.08	0	0	4.54	-0.54
C	5	1.46	3.08	0	0	4.54	-0.54
C	6	1.44	3.18	0	0	4.62	-0.62
C	7	1.43	3.17	0	0	4.6	-0.6
C	8	1.43	3.17	0	0	4.6	-0.6
C	9	1.44	3.17	0	0	4.6	-0.6
Mo	1	2.25	6.55	4.85	0	13.65	0.35
Mo	2	2.28	6.53	4.86	0	13.67	0.33
Mo	3	2.18	6.48	4.91	0	13.57	0.43
Mo	4	2.28	6.53	4.86	0	13.67	0.33
Mo	5	2.25	6.55	4.85	0	13.65	0.35
Mo	6	2.25	6.55	4.85	0	13.65	0.35
Mo	7	2.28	6.53	4.86	0	13.67	0.33
Mo	8	2.24	6.54	4.88	0	13.66	0.34
Mo	9	2.2	6.58	4.88	0	13.66	0.34
Mo	10	2.25	6.68	4.91	0	13.84	0.16
Mo	11	2.2	6.58	4.88	0	13.65	0.35
Mo	12	2.24	6.54	4.89	0	13.66	0.34
Mo	13	2.22	6.55	4.89	0	13.66	0.34
Mo	14	2.24	6.54	4.88	0	13.66	0.34
Mo	15	2.2	6.58	4.88	0	13.66	0.34

(c)

Bond populations for S_C

Bond	Population	Length(Å)	Bond	Population	Length(Å)
C 3 Mo 12	0.64	2.03441	C 8 Mo 12	0.34	2.11443
C 3 Mo 8	0.63	2.04036	C 7 Mo 12	0.34	2.11451
C 3 Mo 14	0.63	2.04045	C 9 Mo 14	0.34	2.11453
C 2 Mo 2	0.53	2.04477	C 7 Mo 14	0.34	2.11458
C 4 Mo 7	0.53	2.04477	C 8 Mo 8	0.34	2.11459
C 4 Mo 4	0.53	2.04605	C 9 Mo 8	0.34	2.11462
C 2 Mo 4	0.53	2.04613	C 1 Mo 7	0.32	2.11832
C 5 Mo 2	0.53	2.04635	C 1 Mo 2	0.32	2.1184
C 5 Mo 7	0.53	2.04643	C 1 Mo 4	0.31	2.11871
C 6 Mo 11	0.36	2.05717	C 9 Mo 10	0.28	2.13068
C 6 Mo 15	0.36	2.06009	C 7 Mo 10	0.28	2.13075
C 6 Mo 9	0.36	2.06017	C 8 Mo 10	0.28	2.13083
C 2 Mo 6	0.42	2.07967	C 1 Mo 1	0.33	2.14064
C 4 Mo 1	0.42	2.07967	C 1 Mo 6	0.33	2.14073
C 4 Mo 5	0.42	2.08149	C 1 Mo 5	0.33	2.14083
C 2 Mo 5	0.42	2.08157	C 9 Mo 13	0.28	2.14121
C 5 Mo 6	0.42	2.08214	C 7 Mo 13	0.28	2.14133
C 5 Mo 1	0.42	2.08223	C 8 Mo 13	0.28	2.14142
C 8 Mo 15	0.37	2.08686	C 5 Mo 3	0.13	2.16837
C 7 Mo 9	0.37	2.08686	C 2 Mo 3	0.13	2.16991
C 8 Mo 11	0.37	2.08729	C 4 Mo 3	0.13	2.17
C 7 Mo 11	0.37	2.08737	C 6 Mo 12	0.26	2.18092
C 9 Mo 9	0.37	2.0875	C 6 Mo 8	0.27	2.18278
C 9 Mo 15	0.37	2.08758	C 6 Mo 14	0.27	2.18288

Figure S5 3D structure model of Mo2C supercell with S_C after optimization (a), as well as atomic population (b) and bond population (c).

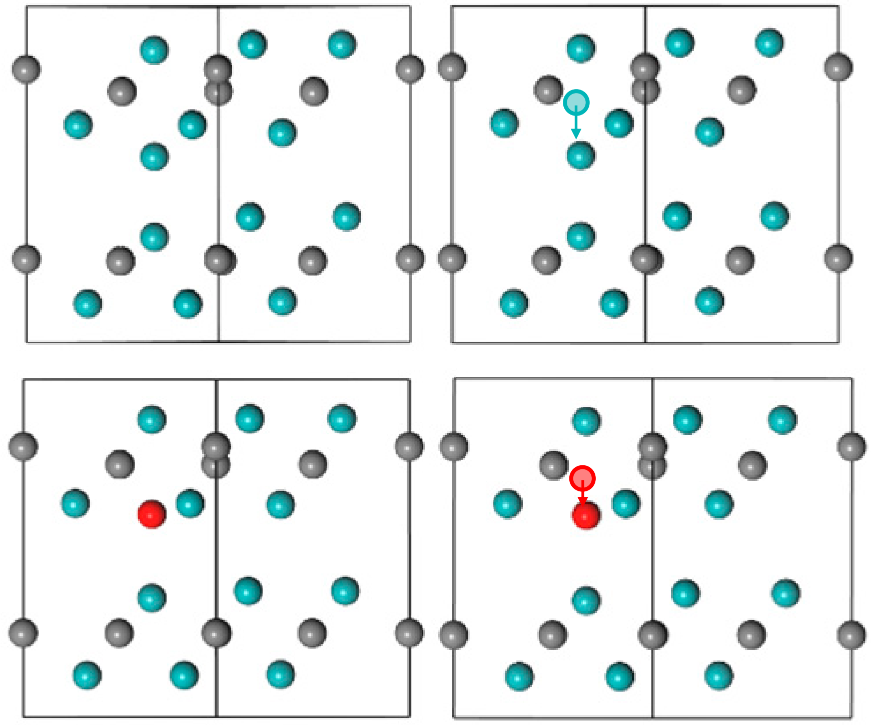

Figure S6 Structure model of Mo2C supercell with interstitial atom after optimization: (a) I_{Mo}(I), (b) I_{Mo}(II), (c) I_V(I) and (d) I_V(II). Semitransparent cyan and red balls represent the original positon for Mo and V atom.

Tables

Table S1 Total energy for different Mo2C supercells with or without vacancy defects and energy for Mo and C atom (eV).

Supercell	$E_{perf}(N)$	$E^{def}_{vacancy}$		E(Mo)	E(C)
		V_{Mo}	V_C		
1×1×1	-4184.059	-2245.430	-4029.102	-1936.860	-155.071
1×1×2	-8368.118	-6427.65	-8212.604	-1936.860	-155.071
1×2×2	-16736.235	-14795.522	-16580.703	-1936.860	-155.071
2×2×2	-32232.884	-31531.833	-33317.045	-1936.860	-155.071
3×2×2	-50208.717	-48268.129	-50053.229	-1936.860	-155.071
3×3×2	-75313.075	-73372.568	-75157.606	-1936.860	-155.071
3×3×3	-112969.601	-111029.074	-112814.06	-1936.860	-155.071

Table S2 Calculated formation energy of vacancy and formation energy of bulk materials with vacancy (eV).

Supercell	$E^{def}_{vacancy}$		$E^{bulk}_{vacancy}$		
	V_{Mo}	V_C	V_{Mo}	V_C	perfect
1×1×1	5.132	0.930	0.239	-0.361	-0.433
1×1×2	5.050	0.402	-0.130	-0.440	-0.437
1×2×2	4.797	-0.088	-0.296	-0.453	-0.430
2×2×2	4.385	-0.880	-0.367	-0.451	-0.436
3×2×2	4.555	-0.726	-0.393	-0.449	-0.436
3×3×2	4.462	-0.803	-0.389	-0.45	-0.435
3×3×3	4.437	-0.907	-0.376	-0.448	-0.436

Table S3 Total energy for Mo2C supercells with substitution and interstitial defects (eV).

Defect type	$E_{perf}(N)$	E^{def}	
		Mo	C
Substation	-32232.884	-34007.186 (S_{Mo})	-30446.863 (S_C)
Interstitial	-32232.884	-34159.492 (I_{Mo}(I,II))	-32384.745 (I_C(I,II))

Table S4 Total energy for Mo2C supercells with V-related defects and energy for V atom (eV).

	$S_{V\text{-}Mo}$	$S_{V\text{-}C}$	I_V(I)	I_V(II)
E^{def}	-32273.271	-34048.676	34202.379	34201.550
